# Safety and Efficacy of Thermal Ablation for Small Renal Masses in Solitary Kidney: Evidence from Meta-Analysis of Comparative Studies

**DOI:** 10.1371/journal.pone.0131290

**Published:** 2015-06-29

**Authors:** Quancheng Yang, Fanzheng Meng, Kai Li, Tong Wang, Qingyuan Nie, Zi Che, Min Liu, Yan Sun, Lin Zhao

**Affiliations:** 1 Department of Urology, Central Hospital of Zibo, Zibo, China; 2 Department of Neurology, Tianjin Nankai Hospital, Tianjin Medical University, Tianjin, China; 3 Department of Urology, Tianjin Third Central Hospital, Tianjin, China; 4 Department of Pharmacy, Central Hospital of Zibo, Zibo, China; 5 International Medical School, Tianjin Medical University, Tianjin, China; ISMETT-UPMC Italy/ University of Catania, ITALY

## Abstract

**Objective:**

To evaluate comparative renal functional preservation, perioperative and oncologic outcomes, and complications of thermal ablation (TA) versus partial nephrectomy (PN) in management of Small renal masses (SRMs) in solitary kidney.

**Methods and Findings:**

Medline, Embase, Web of Science and the Cochrane Library were systematically searched. A meta-analysis for comparative studies comparing TA with PN was performed. According to predefined inclusion criteria, seven datasets were identified from 8 observational studies including a total of 628 patients. Cumulated data showed the changes of creatinine (*p*=0.02) and estimated glomerular filtration rate (eGFR) (*p*<0.0001) in TA arm were significantly less than these in PN arm. Significantly less new-set chronic kidney disease (CKD) was observed in TA group (*p*=0.04). In terms of postoperative dialysis rate, the difference favoring TA was also noted, though there is no statistical significance (*p*=0.09). With regard to perioperative outcomes, our data demonstrated that patients who underwent TA had significantly shorter operation time (*p*=0.002), less blood loss (*p*<0.0001), shorter length of stay (*p*<0.00001), and less transfusion rate (*p*=0.01) than those underwent PN. In addition, patients underwent TA suffered less intra- and postoperative complications (*p*=0.007, *p*<0.00001; respectively). With regard to oncologic outcomes, disease-free survival (DFS) (*p*<0.00001) and cancer-specific survival (CSS) (*p*=0.01) in the PN arm were significantly better than these of the TA arm. But, TA yielded a comparable overall survival to PN (*p*=0.40). Sensitivity analyses led to very similar results with overall results, and confirmed its stability.

**Conclusions:**

Our analysis indicates that PN have advantage in controlling cancer recurrence. However, TA is associated with significantly better renal functional preservation and perioperative outcomes, and less complications without increasing overall death. Our data suggest that indication for TA may be extended to select younger, healthier patients who desire a much less invasive therapeutic option.

## Introduction

Increased use of abdominal imaging has led to an increasing diagnosis of small renal masses (SRMs) presenting with clinically local-confined disease [1]. These masses are very heterogeneous, with approximately 20% of SRMs smaller than 4 cm on the preoperative imaging examination are benign, and only about 20–25% exhibiting potentially aggressive cancer after the diagnosis [2,3]. To treat patients with SRMs, three competing factors have to be balanced: cancer control, competing comorbidities, and renal functional outcomes.

According to the American Urological Association (AUA) and European Association of Urology (EAU) guidelines, thermal ablation (TA) represents a better option for patients with significant comorbidities who are unfit for surgery [3,4,5]. A systematic review [6] comparing laparoscopic cryoablation (CA) to PN was published, which reported a significant increase in local progression with CA. However, there is no adequately comparable control group evaluating effects and safety of CA. For the management of SRMs in patients with an anatomically or functionally solitary kidney, preserving renal function is imperative to be taken into account. Because, patients with significant losses in renal function may develop chronic kidney disease (CKD) with attendant cardiovascular risks and increased mortality [7,8]. As a result, PN turns into the generally recommended procedure in the case of insufficient evidence supporting much less invasive treatment, such as thermal ablation (TA). PN generally requires renal hilar clamping with warm ischemia, which may have deleterious effects on renal function. However, no guideline exists to recommend available alternative of PN for management of SRMs in the presence of a solitary kidney.

In recent years, a number of studies have been published in an attempt to explore if the TA is an effective and safe substitution for treatment of SRMs in solitary kidney, but the results are inconsistent [9–16]. In addition, small sample size at single-centre prevents credible conclusions from being obtained. Therefore, this systematic review and meta-analysis of comparative studies was performed.

## Evidence Acquisition

### Search strategy and selection criteria

We conducted a comprehensive literature review by searching Medline, Embase, Web of Science and the Cochrane Library using predefined search terms (S1 File) without language and date restriction. We also reviewed the reference lists of relevant publications. Only full-text articles published in peer-reviewed journals were identified. We included studies according to the following criteria: 1) Patients with functional or anatomic solitary kidney were evaluated. 2) Comparative data were available. Cases were treated with CA, RFA or other ablation techniques, while subjects from control group were treated with methods of PN or enucleation. 3) Data for dichotomous and/or continuous variables must be directly provided or calculable from the data source. 4) When some studies with the same population were identified, all of them were assessed carefully to ensure no useful information was missed, so more than one of them might be included together.

### Data extraction and presentation

This study were conducted according to the Preferred Reporting Items for Systematic Reviews and Meta-Analyses (PRISMA) [17] and Meta-analysis of Observational Studies in Epidemiology (MOOSE) [18] guidelines. Two investigators (Wang and Nie) independently extracted data, and disagreements were resolved through discussion with the third author (Yang). The listed study characteristics were extracted: study design, numbers of subjects, gender, age, comorbidity, American Society of Anesthesiologists (ASA) score, pre- and postoperative renal function, and the side, size and pathology of masses. If necessary, authors were contacted for requesting additional data. Quality assessment was undertaken independently by two authors (Che and Liu). The methodological quality of the includued studies was assessed by the modified Newcastle-Ottawa scale (mNOS) [19], which contains three elements: selection of subjects, comparability of the study groups, and assessment of outcome (S2 File). A score of 0–9 was assigned to each study, and observational studies achieving more than five stars were considered to be of high quality.

The main endpoint of this study was renal functional preservation. Four measurements on renal function were evaluated including changes of creatinine and percent of estimated glomerular filtration rate (eGFR), new-set chronic kidney disease (CKD) and postoperative dialysis rate (percent of patients need dialysis after treatment). We take into account all patients requiring temporary or persistent dialysis. Subgroup analysis for persistent dialysis rate wasn’t addressed because of insufficient data. The secondary endpoints were perioperative outcomes (e.g., operative time, length of stay [LOS], estimated blood loss [EBL], and transfusion and conversion rate), complications (intraoperative and postoperative complications within 30 d of surgery) and oncologic outcomes (local recurrence rate, metastasis rate, recurrence-free survival [RFS], cancer-specific survival [CSS], and overall survival [OS]). If data were available, postoperative complications were subdivided into major and minor complications. Postoperative complications were classified according to the Clavien classification system [20]. Minor complications consisted of grade 1 and grade 2, and major complications included grades 3–5. Local recurrence was imageologically defined as tumor relapsed or newly observed in kidney during the follow-up period.

### Statistical analysis

For continuous data, inverse variance of χ^2^ was used and expressed as mean difference (MD) with 95% confidence interval (CI). For dichotomous variables, hazard ratio (HR) taking into account of time variable, was prioritized and analyzed with method of inverse variance. The simplest method collecting HRs is to gather them from original article directly. If direct data were not available, the numbers of events and patients at risk in each group, and the log rank statistic or its *P* value allowing estimating HRs. If data were only provided in the form of survival curve, we extracted survival rates from some specified time so as to estimate HRs and the corresponding variances [21]. Sensitivity analyses were performed for high-quality studies to test the stability of cumulated results. Funnel plots were used to screen for potential publication bias.

Fixed-effects model, assumed that all samples came from populations with the same effect size, was used when heterogeneity was absent; otherwise, random-effects model was used. Heterogeneity was assessed by Q test [22]. We also calculated the *I* ^2^ statistic that represented percentage of total variation across studies. As a guide, *I* ^2^ values of 25%, 50%, and 75% were considered as indication of low, medium, and high levels of heterogeneity [23]. All analyses were conducted by using Review Manage, version 5.2 (The Cochrane Collaboration, Oxford, U.K.). All *P* values were two-sided and considered statistically significant when less than 0.05.

## Evidence synthesis

### Literature search and characteristics of studies

**Fig 1:**
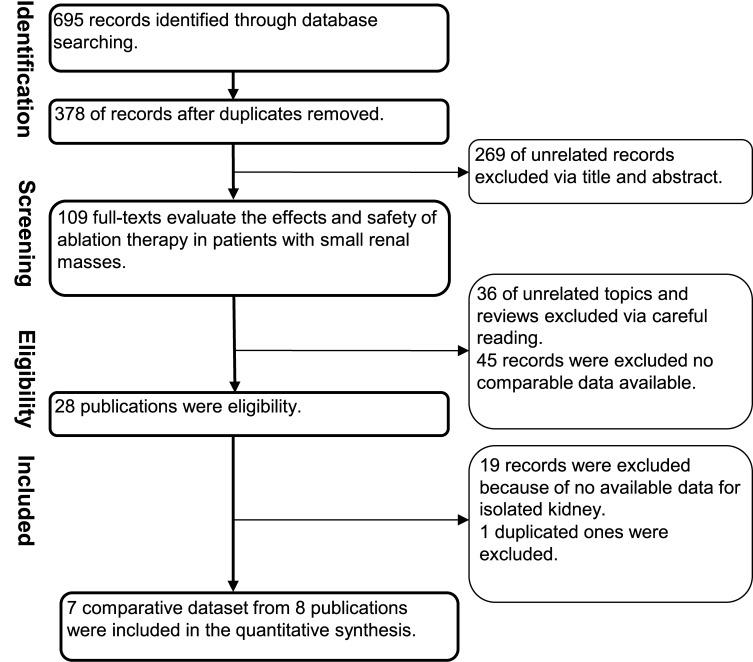
Flow diagram of studies identified, included, and excluded.

We screened 378 potential, non-duplicate articles. In light of titles and abstracts, 109 papers were got with full texts might be of potential interest. After reading through full-texts, 28 studies that appeared to meet the inclusion criteria were transmitted to further examination. Among them, 19 records were excluded because bilateral kidney was included (S3 File). At the last, 8 studies [9–16] reporting on 7 datasets were included with 324 cases and 304 controls (Fig 1). We suspected that four articles [10,14,16,24] had overlapping subjects but three of them [10,14,16] presented data on different outcomes, therefore, we only excluded one [24]. In addition, one article reported two separate datasets, one for CA versus LPN (named **a**) and one for RFA versus LPN (named **b**) [16].

The detailed characteristics of included studies were shown in Table 1. All studies were published in the last three years, reflecting the recent realization of using TA in selective patients with solitary kidney. Study qualities were rated based on mNOS, only one study reached less than six stars and was identified as of low quality. After eliminating this study, sensitivity analysis was performed to confirm our findings. The methodological qualities of included studies were listed in S1 Table.

**Table 1:** Baseline characteristics of included studies.

Study	Design,	No. of cases,	Etiology*	Preoperative renal function			Pathology	ASA^¶^	Age, yr^†^	Tumor Size,	Follow-up, mo^†^	Quality
	LOE	type	Co/At/Ma/Be	Creatinine^†^	eGFR^†^	CKD (%)	Ma/Be/Un			cm^†^		Score^§^
Mues 2012 [1]	C-C, 3b	98 AT	NA	1.4	59	NA	69/6/23	NA	64 (38–86)	2.5 (1–4.4)	31	7
		100 PN		1.4	59		97/3/0		64 (35–92)	3.9 (1–10)	24	
Kamol 2013 [2]	Cohort, 2b	43 CA	2/1/39/1	1.3 (1.2–1.5)	57 (51–63)	69.8	25/13/5	83%	64 (57–72)	2.2 (1.6–3.2)	41 (26–59)	6
		33LPN	2/4/24/3	1.2 (1–1.4)	62 (51–73)	42.4	24/9/0	59%	60 (51–69)	2.9 (1.8–4.2)	17 (5–62)	
Turna 2009 [4]	P-C, 2b	36 CA	2/4/27/3	1.4±0.5	52.3±19.7	72.2	22/8/6	78%	64.1 (35–83)	2.5 (1.1–5.0)	24.0 (1–84)	6
		29 PRFA	3/1/23/2	1.4±0.5	52.3±16.2	58.6	24/5/1	69%	60.7 (30–87)	2.6 (0.9–4.2)	14.0 (1–44)	
		36 LPN	3/5/24/4	1.4±0.4	65±23.5	63.9	23/13/0	67%	60.3 (20–87)	3.7 (1.4–10.7)	42.5 (7–81)	
Goyal 2011 [5]	Cohort, 2b	23 CA	NA	1.3±0.4	54.6±16.5	65.2	NA	NA	68 (40–79)	2.5 (1–4)	31.2 (0.6–153)	7
		15 PN		1.5±0.7	55.07±22.2	60.0			65 (47–85)	3.4 (1–5.5)	30.8 (0.1–113.5)	
Mitchell 2011 [6]	Cohort, 2b	50 PAT	NA	1.3 (0.8–2.6)	53.4 (25.5–80.9)	68.0	NA	76%	63 (27–83)	2.5 (1.2–7.3)	NA	6
		62 OPN		1.5 (0.7–3.3)	53.5 (19.7–123.7)	72.6		63%	69 (49–89)	3.5 (0.7–13.0)		
Raman 2009 [7]	C-S, 4	47 RFA	NA	NA	46.5 (15.9–91.6)	85.1	40/5/8	NA	65.9±16.7	2.7 (1.5–6.5)	18.1 (6–66)	4
		42 OPN			55.9 (30.5–89.7)	52.4	37/9/0		59.6±12.8	3.5 (1.3–5.5)	30.0 (11–83)	
Olweny 2012 [8]	Cohort, 2b	37 RFA	NA	NA	NA	NA	37/0/0	95%	64 (56–69)	2.1 (1.8–2.8)	78.0 (69.6–85.2)	6
		37 PN					37/0/0	76%	55 (48–59)	2.5 (1.7–3.1)	73.2 (64.8–87.6)	
Haber 2011 [9]	P-C, 2b	30 LCA	0/4/28/1	1.5±0.5	53.8±19.0	NA	25/5/0	2.7±0.8	60.9±11.4	2.6±1.08	60.2±46.3	7
		48 LPN	4/8/31/5	1.2±0.3	61.6±18.6		36/12/0	2.7±0.5	60.6±13.7	3.2±1.33	42.7±30.8	

LOE = Level of evidence; C-C = case-control; P-C = prospective cohort; C-S = case-series; AT = ablation therapy; PN = partial nephrectomy; CA = cryoablation; LPN = laparoscopic partial nephrectomy; PRFA = percutaneous radiofrequency ablation; PAT = percutaneous ablative therapy; OPN = open partial nephrectomy; RFA = radiofrequency ablation; NA = not applicable; eGFR = estimated glomerular filtration rate (ml/min/1.73m2); CKD = chronic kidney disease; Ma/Be/Un = malignant/benigh/unknown.

* Etiology of solitary kidney; Co/At/Ma/Be means isolated kidney was caused by congenital, atrophic, malignancy or benign disease.

^†^ Mean or median.

^¶^ Percent of patients with American society of anesthesiologists (ASA) score 3 or 4; in study by Haber et al, ASA score is showed as mean ± standard difference.

^§^ Modified Newcastle-Ottawa scale.

### Renal functional outcomes

**Fig 2:**
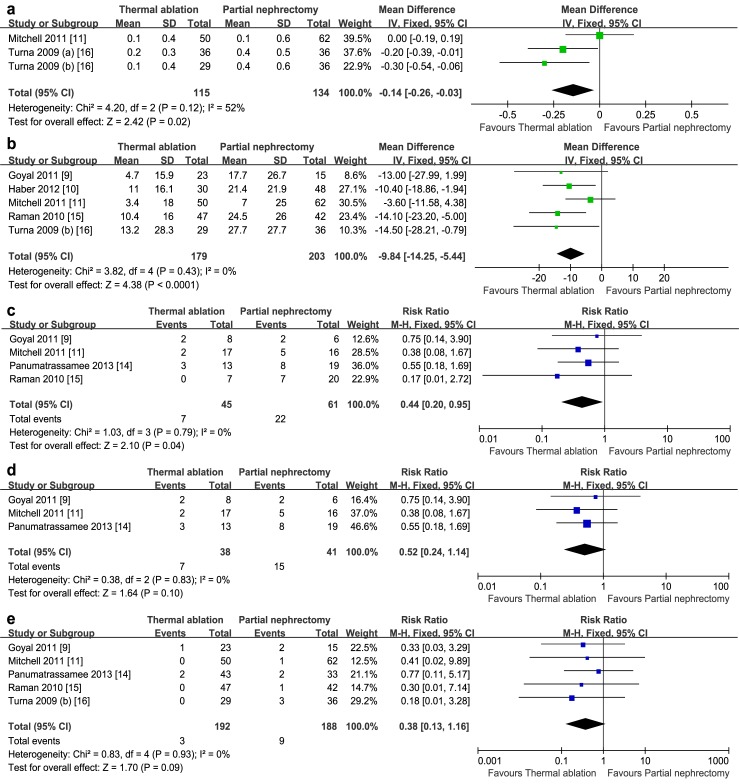
Forest plot and meta-analysis of renal functional outcomes: a) Increase of creatinine; b) Decrease of eGFR; c) Risk of new-set chronic kidney disease (CKD); d) Sensitivity analysis for risk of new-set CKD; e) Postoperative dialysis rate. IV = inverse variance method; M-H = Mantel-Haenszel method; CI = confidence interval.

Patients with worse renal function might be likely selected to be treated with TA [14,15]. So, one-point (such as postoperative) creatinine and eGFR may bias us against TA when evaluating renal functional outcomes. As a result, the increase of creatinine, decrease of eGFR, incidence of new-set CKD and postoperative dialysis rate were cumulated in this analysis. Results showed that the increase of creatinine (MD: -0.14; 95% CI, -0.26 to -0.03; *p* = 0.02; Fig 2a) and decrease of eGFR (MD: -9.84; 95% CI, -14.25 to -5.44; *p*<0.0001; Fig 2b) in the TA arm were significantly less than these in PN arm. Remarkably fewer new-set CKD was also observed in TA group (RR: 0.44; 95% CI, 0.20–0.95; *p* = 0.04; Fig 2c). For postoperative dialysis rate, a non-statistical difference favoring TA was noted (RR: 0.38; 95% CI, 0.13–1.16; *p* = 0.09; Fig 2e). We failed to find any notable heterogeneity between studies (*p* = 0.12, *I* ^*2*^ = 52%; *p* = 0.43, *I* ^*2*^ = 0%; *p* = 0.79, *I* ^*2*^ = 0%; *p* = 0.93, *I* ^*2*^ = 0%; respectively), which shows favorable internal consistency among included studies.

### Perioperative outcomes

Compared to PN, patients underwent TA had significantly shorter operation time (MD: -26.32; 95% CI, -43.05 to -9.59; *p* = 0.002; S1 Fig), less EBL (MD: -294.58; 95% CI, -431.60 to -157.57; *p*<0.0001; S1 Fig) and shorter LOS (MD: -2.25; 95% CI, -2.70 to -1.79; *p*<0.00001; S1 Fig). According to the cumulative data of published studies, transfusion rates were 1.0% (2 of 193 patients) in TA group and 6.5% (12 of 184 patients) in PN group. Pooled result revealed that transfusion rate was 3.35 times in TA less than in PN group (RR: 0.23; 95% CI, 0.08–0.72; *p* = 0.01; S1 Fig). With regard to convention rate, pooled result only showed a trend favoring TA without significance (RR: 0.18; 95% CI, 0.03–1.01; *p* = 0.05; S1 Fig). No significance of heterogeneity was found in this group of analyses, so, fixed-effects models were used assuming that studies were sampled from populations with the same effect size.

### Complications

In terms of complications, pooled data showed significant lower incidences of intra- and postoperative complications in patients underwent TA (RR: 0.16, 95% CI: 0.04–0.60, *p* = 0.007; RR: 0.27, 95% CI: 0.16–0.43, *p*<0.00001, S2 Fig). Then the postoperative complications were subdivided to major (RR: 0.33; 95% CI, 0.13–0.89; *p* = 0.03; S2 Fig) and minor (RR: 0.38; 95% CI, 0.16–0.88; *p* = 0.02; S2 Fig) ones, the significant advantages of TA were also observed in both subgroup-analyses. Again, there was no remarkable heterogeneity between studies.

### Oncologic outcomes

In cumulative analysis of studies reporting data on local recurrence and metastasis rates, no significant difference between TA and PN was observed (RR: 2.37, 95% CI: 0.74–7.56, *p* = 0.15; RR: 1.87, 95% CI: 0.81–4.33, *p* = 0.14, S3 Fig). With regard to the survival of patients with localized RCC, HRs were collected from individual studies, which took into account the time variable. Pooled data showed that CSS (HR: 9.00; 95% CI, 1.63–49.71; *p* = 0.01; S3 Fig) and DFS (HR: 12.12; 95% CI, 4.35–33.75; *p*<0.00001; S3 Fig) were significantly better in PN arm. Estimate of OS in patients treated with TA was in the same direction with other survival data but did not reach significance (HR: 1.33; 95% CI, 0.68–2.59; *p* = 0.40; S3 Fig). Significant heterogeneity was only found in analysis for local recurrence rate (*p* = 0.04, *I* ^*2*^ = 57%). In this analysis, two datasets from the Cleveland Clinic used procedure failure as a substitute for local recurrence rate [14,16]. After removing the two datasets, between-study heterogeneity disappeared (*p* = 0.50, *I* ^*2*^ = 0%), and the non-significantly different recurrence rate between the two groups was persistent (RR: 1.37; 95% CI, 0.64–2.94; *p* = 0.41).

### Sensitivity analysis and publication bias

Six high-quality retrospective studies [9–14,16] scored more than 5 stars on the mNOS were included in the sensitivity analyses, which led to very similar results with overall analyses. Only one change in the significance was observed in the cumulated outcomes evaluating incidence of new-set CKD (Fig 2d). Nevertheless, trend favoring TA still existed (RR: 0.52; 95% CI, 0.24–1.14; *p* = 0.10). In our assessment of publication bias, funnel plots showed balance, with points distributing symmetrically, indicating no obvious publication bias existed (S4 Fig).

## Discussion

Because of the rare incidence of SRMs in solitary kidney, we failed to carry out a prospective study with long term follow up to compare TA with PN concerning renal functional preservation, morbidity, and cancer control. Then this meta-analysis based on seven observational studies involving 324 cases and 304 controls is conducted. Our analysis indicates that PN have advantage in controlling cancer recurrence. However, TA is associated with significantly better renal functional preservation and perioperative outcomes and fewer complications without increasing overall death. More kidney cancer–related deaths in TA arm tended to be balanced by more deaths not related to kidney cancer in PN arm.

### Renal function

Solitary kidney is one of the most significant risk factor of renal failure after nephron-sparing surgery (NSS) [25], and decrease in renal function independently predicted for an increase in hospitalization, cardiovascular events, and mortality [8], so maximally preserving renal function assumes very important while making choice of management of SRMs in patients with solitary kidney. In an observational study comparing Laparoscopic (LPN) to open partial nephrectomy (OPN) in management of tumors in solitary kidney, longer warm ischemia time (more than 20 minutes) was recognized as independent risk factors predicting poor postoperative eGFR [26]. Then cold ischemia in PN, as well as TA was introduced to improve the probability of preserving remaining renal function. In series of 84 patients undergoing PN [27], cold ischemia was found to be associated with immediate eGFR decreases (*p* = 0.008). Beyond 1 month after surgery, significantly advantage of cold ischemia in lessening the change of eGFR was disappeared. Additional one observational cohort study was performed in multicenter for tumor in solitary functioning kidney under cold (300 cases) or warm (360 cases) ischemia [28]. Results also showed similar rates of eGFR decrease three months after surgery in the two groups.

For advantage of TA in protecting renal function against loss, Raman et al. [15] presented outcomes after NSS in a series of 89 patients with a solitary kidney. The eGFR at 12 months decreased by 24.5% and 10.4% in RFA and PN groups, respectively. The advantage of TA in protecting renal function was confirmed by a cohort 78 patients with small tumor (clinical stage T1) in solitary kidney [10]. By three months after treatment, eGFR decreased by 21.4% in PN group and 11% in CA group (*p* = 0.02). In this study, results also revealed that more patients in the PN group had a CKD upgrade of more than one stage compared to CA (39.5% vs. 13.3%, *p* = 0.01) [10]. Our analyses reconfirmed that TA was much more effective in preventing change of creatinine and eGFR than PN. Evidences listed above suggest that TA is a reasonable treatment approach in patients with poor renal function or those at risk of CKD including those with a solitary kidney.

In terms of new-set CKD, we found a significant advantage of TA in preventing its development. Sensitivity analysis of high-quality studies also indicated an obvious a trend favoring TA, but it didn’t reach significance. It’s likely representing inadequate power to detect significant difference in a rare event with such small sample sizes. So, additional studies are needed to confirm this result.

### Perioperative outcomes and complications

Patients undergoing TA had significantly shorter operative times, less blood loss, shorter length of stay, and lower transfusion and conversion rate compared to those underwent PN. Moreover, ablative techniques companied with lower complication rates (including intra- and postoperative ones). In our analysis, there was no sufficient data to distinguish laparoscopic TA from percutaneous method. TA with percutaneous is much less invasive than laparoscopic, indicating mixed techniques in our study may misestimate the cumulated analyses on perioperative outcomes and complications. In a cohort of 78 patients with a small tumor in a functionally solitary kidney, the study was restricted to compare laparoscopic procedures (LPN vs. laparoscopic cryoablation [LCA]) [10]. Results confirmed that LPN was significantly associated with greater blood loss (391 versus 162 ml; P = 0.003), and trended towards more post-operative complications (22.9% vs. 6.7%; P = 0.07). In another cohort comparing LPN with percutaneous RFA for SRMs in solitary kidney, more intraoperative complications (13.9% vs. 0%) and postoperative complications (58.3% vs. 7%) were also noted in LPN group [16]. These advantages of TA in lessening complications and improving perioperative outcomes, either percutaneous or laparoscopic, supports it to be an available treatment option for the patient at high surgical risk who wants active treatment.

### Oncologic outcomes

In the present analyses, data showed that DFS in the PN arm was significantly better than that in TA. That doesn’t mean TA should be abandoned. Unlike PN, TA needs not to be offered to patients only once. SRMs are allowed to be re-ablation without increasing operative difficulty [29–31]. In the series of 40 patients underwent RFA [31], Stern et al reported two recurrences in 40 patients, the first one was safely re-ablated and the patient was cancer-free at 30 months from initial ablation. The other one with incomplete tumor was re-ablated and the patient was tumor-free 42 months after initial ablation. It suggests that recurrent tumor can be managed in time if active surveillance is effectively carried out in patients treated by TA.

For survival rates, we found that CSS was significantly better in the PN group, while OS was comparable between the two groups. In these analyses, only one included studies reported individuals with median follow-up less than five years [16]. After removing this study, persistently different CSS favoring PN (HR: 10.08; 95% CI, 1.21–84.25; *p* = 0.03) and equivalent OS (HR: 1.25; 95% CI, 0.59–2.66; *p* = 0.56) were revealed. There is no surprise since the complex parameter of OS, which was hard to predict. A reason that can’t be neglected is that renal function significantly deteriorates in patients treated by PN. These patients may develop chronic kidney disease (CKD) with attendant cardiovascular risks and increased mortality [7,8]. Based on that the evidence supporting an equivalent OS between TA and PN, our data suggests that TA is an effective substitute on improving overall survival of patients with anatomically or functionally solitary kidney. The promised survival rate suggests that indication for TA may not be limited to select patients with significant comorbidities who were poor surgical candidates but yet desired active treatment.

Some limitations in evaluating oncologic outcomes are needed to be addressed in further prospective studies with additional populations. Firstly, it is reported that only 78% of resected SRMs smaller than 4 cm on the preoperative imaging examination are malignant [2]. In this analysis, we failed to include selective patients with diagnostic renal cell carcinoma (RCC). It means that effect of TA on cancer control may be over- or underestimated because the remained 22% benign masses don’t need any kind of operation. To define histology, core biopsy is recommended to be performed prior to treatment (Grade B) [4]. In addition, previous meta-analysis suggested that CA was better than RFA in controlling local progression of renal tumors [32]. However, subgroup analysis concerning cancer control stratified by technique of TA was not performed because of insufficient data.

### Strengths and limitations

Sensitivity analyses were performed to re-evaluate the effect estimates with only high-quality studies included. As expected, the results weren’t overturned. To minimize potential publication bias, we conducted our research without language restriction. In addition, obtained funnel plot kept equilibrium, indicating no obvious publication bias. In a word, this work integrated data from studies accord to predefined criteria; thus, we have confidence on our findings.

However, this meta-analysis has a few limitations that should be noted. Firstly, all included studies were retrospective, which may introduce selection bias. Patients received TA tend to be accompanied with significant comorbidities [33], indicating that observed better renal function preservation and less complications in TA arm might be weakened. Nevertheless, small exophytic tumors have tended to be treated with TA while large and complex tumors are usually managed with PN [33], which may causes overestimating the advantages of TA on complications and perioperative outcomes. Unfortunately, data to stratify tumors by complexity were insufficient for analysis. Secondly, sample sizes of included studies are small in this meta-analysis, which is a reflection of the rarity of SRMs in solitary kidney. Finally, the follow-up periods were generally short and varietal between the comparative groups, so outcomes with identical and long term follow-up, especially for oncologic and renal functional outcomes are expected.

## Conclusions

For patients with a solitary kidney, PN and TA are both safe and effective treatment options. PN is superior to TA in controlling tumor progression. Compared to PN, TA is associated with better renal functional maintenance, shorter operation time, less EBL, shorter LOS, and less intra- and postoperative complications. As an effective minimally invasive therapy, TA yields an equivalent long-term OS to PN. These results indicate that TA is safe and effective.

## Supporting Information

S1 Fig Forest plot and meta-analysis of perioperative outcomes: a) Operation time; b) Estimated blood loss; c) Length of stay; d) Transfusion rate; e) Conversion rate. IV = inverse variance method; M-H = Mantel-Haenszel method; CI = confidence interval.
(TIF)

S2 Fig Forest plot and meta-analysis of complication rates: a) Intraoperative complication rate; b) Postoperative complication rate; c) Postoperative complication rate (Major); d) Postoperative complication rate (Minor). M-H = Mantel-Haenszel method; CI = confidence interval.
(TIF)

S3 Fig Forest plot and meta-analysis of oncologic outcomes: a) Local recurrence rate; b) Metastasis rate; c) Overall survival; d) Cancer-specific survival; e) Disease-free survival. M-H = Mantel-Haenszel method; CI = confidence interval; IV = inverse variance method.
(TIF)

S4 Fig Funnel plots illustrating meta-analysis of renal function outcomes (a), oncologic outcomes (b), perioperative outcomes (c), and complications (d). SE = standard error; SMD = standard mean difference; RR = risk ratio.
(TIF)

S1 File Predefined search terms, according which the comprehensive literature review was conducted. (DOC)

S2 File The methodological quality of the includued studies was assessed by the modified Newcastle-Ottawa scale (mNOS), containg three elements: selection of subjects, comparability of the study groups, and assessment of outcome. (DOC)

S3 File The list of excluded because of bilateral kidney. (DOC)

S1 Table Risk of bias in retrospective studies using modified Newcastle-Ottawa scale. (DOC)

S1 PRISMA Checklist PRISMA checklist. (DOC)
