# Supplementary figures and images for: Safety and Efficacy of Thermal Ablation for Small Renal Masses in Solitary Kidney: Evidence from Meta-Analysis of Comparative Studies
Source: PLoS One. 2015 Jun 29;10(6):e0131290. doi: 10.1371/journal.pone.0131290 (PMC4484808; doi:10.1371/journal.pone.0131290)

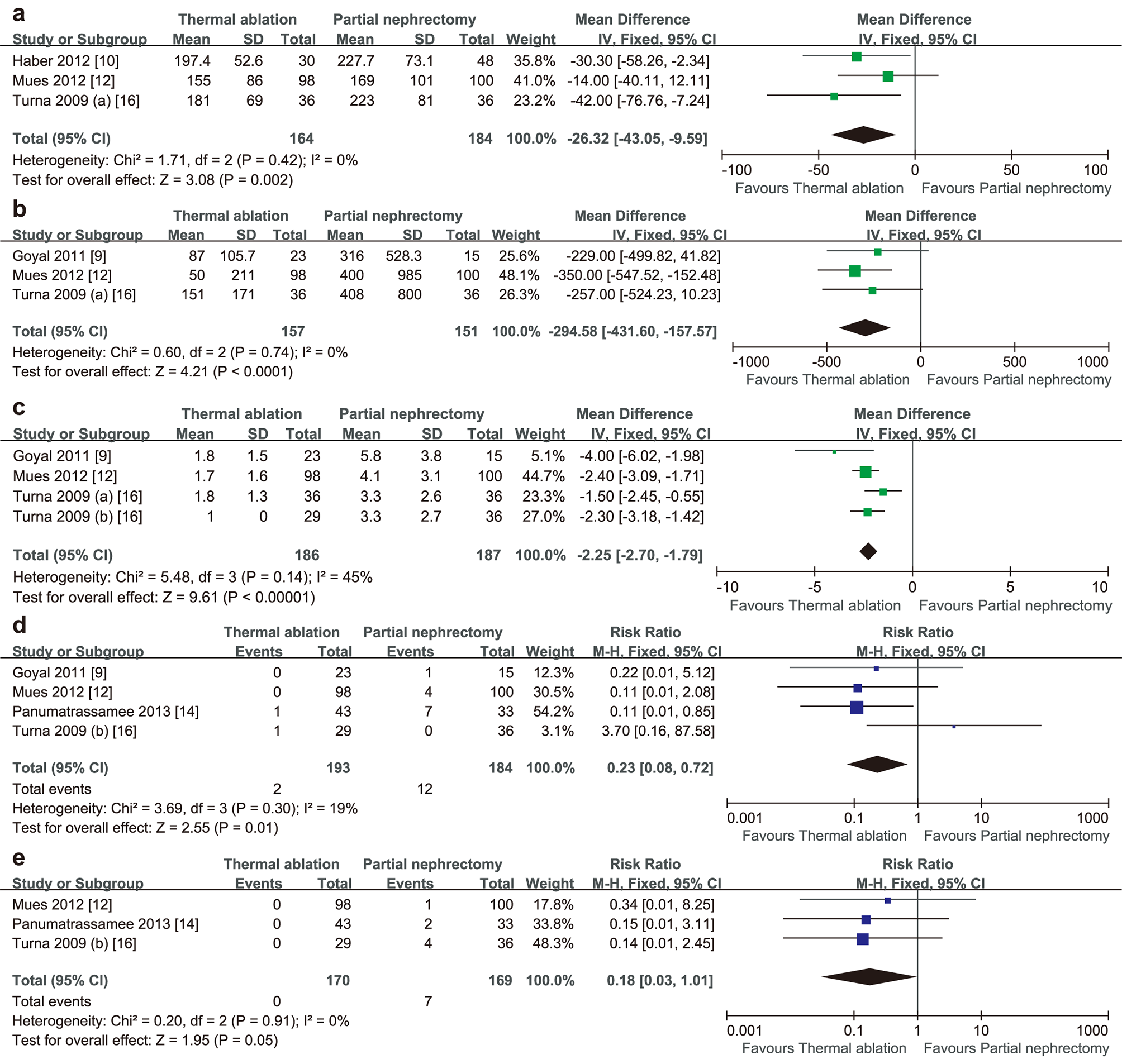

Supplement: S1 Fig — Forest plot and meta-analysis of perioperative outcomes: a) Operation time; b) Estimated blood loss; c) Length of stay; d) Transfusion rate; e) Conversion rate. IV = inverse variance method; M-H = Mantel-Haenszel method; CI = confidence interval. (TIF) [file pone.0131290.s001.tif]

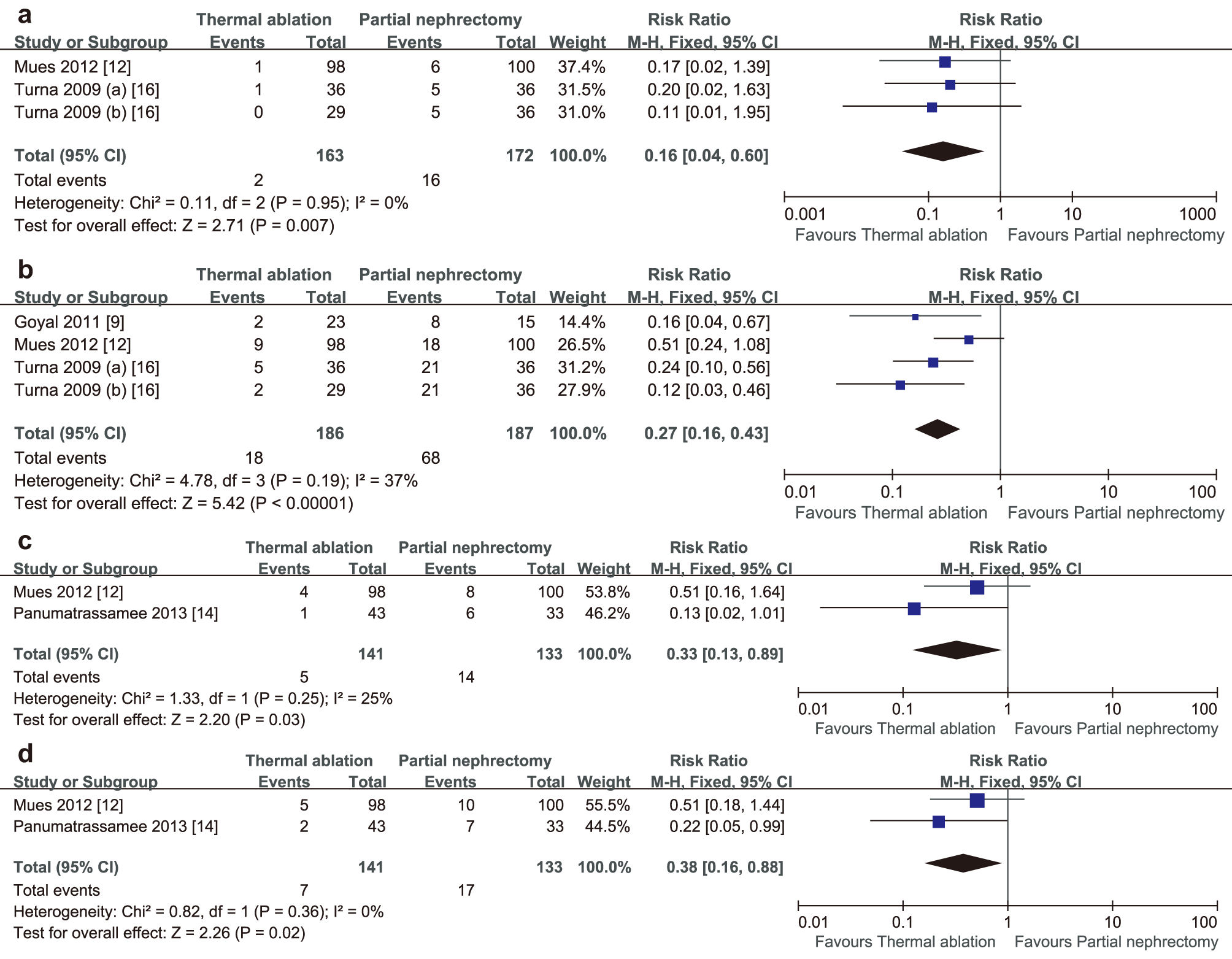

Supplement: S2 Fig — Forest plot and meta-analysis of complication rates: a) Intraoperative complication rate; b) Postoperative complication rate; c) Postoperative complication rate (Major); d) Postoperative complication rate (Minor). M-H = Mantel-Haenszel method; CI = confidence interval. (TIF) [file pone.0131290.s002.tif]

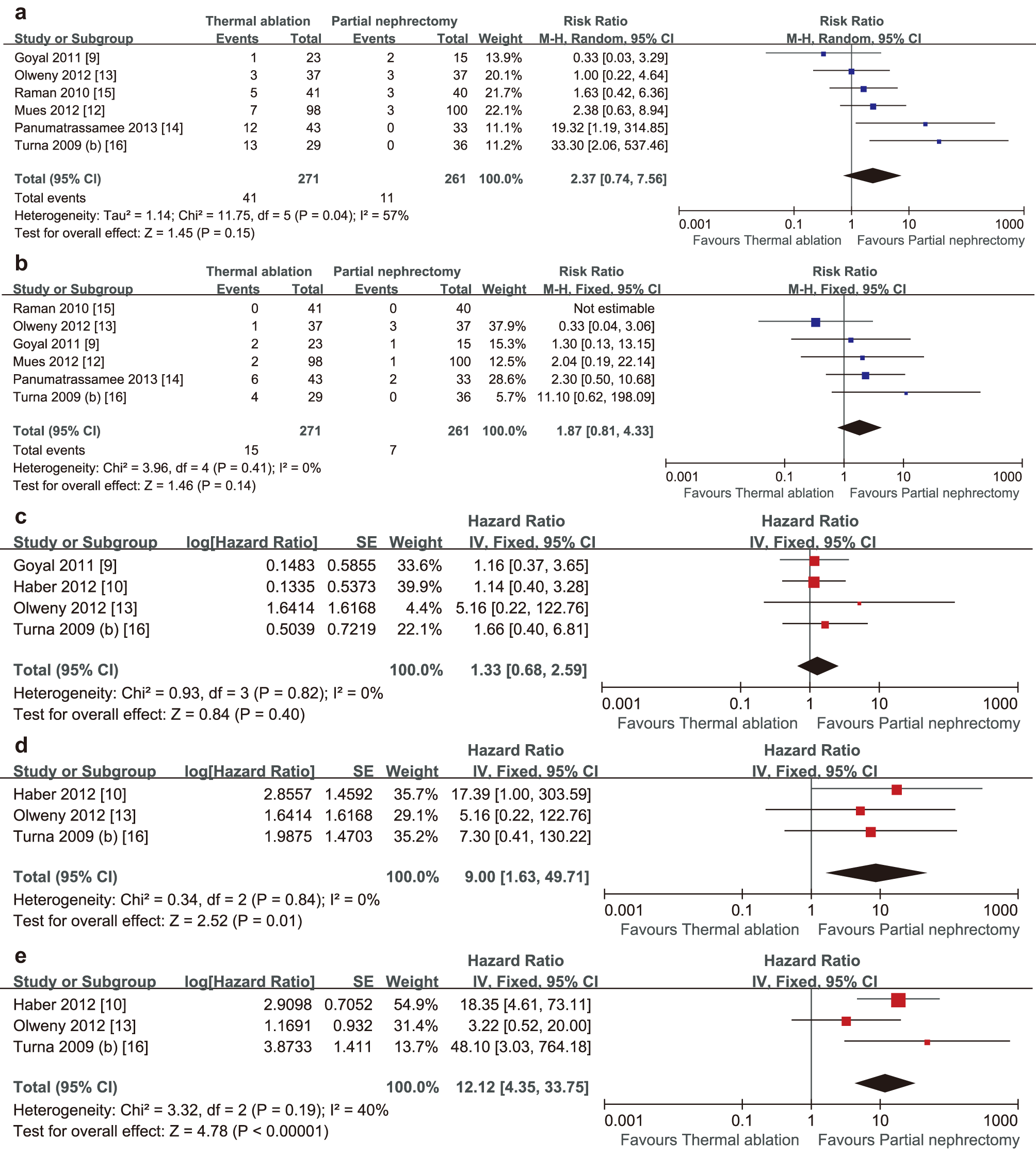

Supplement: S3 Fig — Forest plot and meta-analysis of oncologic outcomes: a) Local recurrence rate; b) Metastasis rate; c) Overall survival; d) Cancer-specific survival; e) Disease-free survival. M-H = Mantel-Haenszel method; CI = confidence interval; IV = inverse variance method. (TIF) [file pone.0131290.s003.tif]

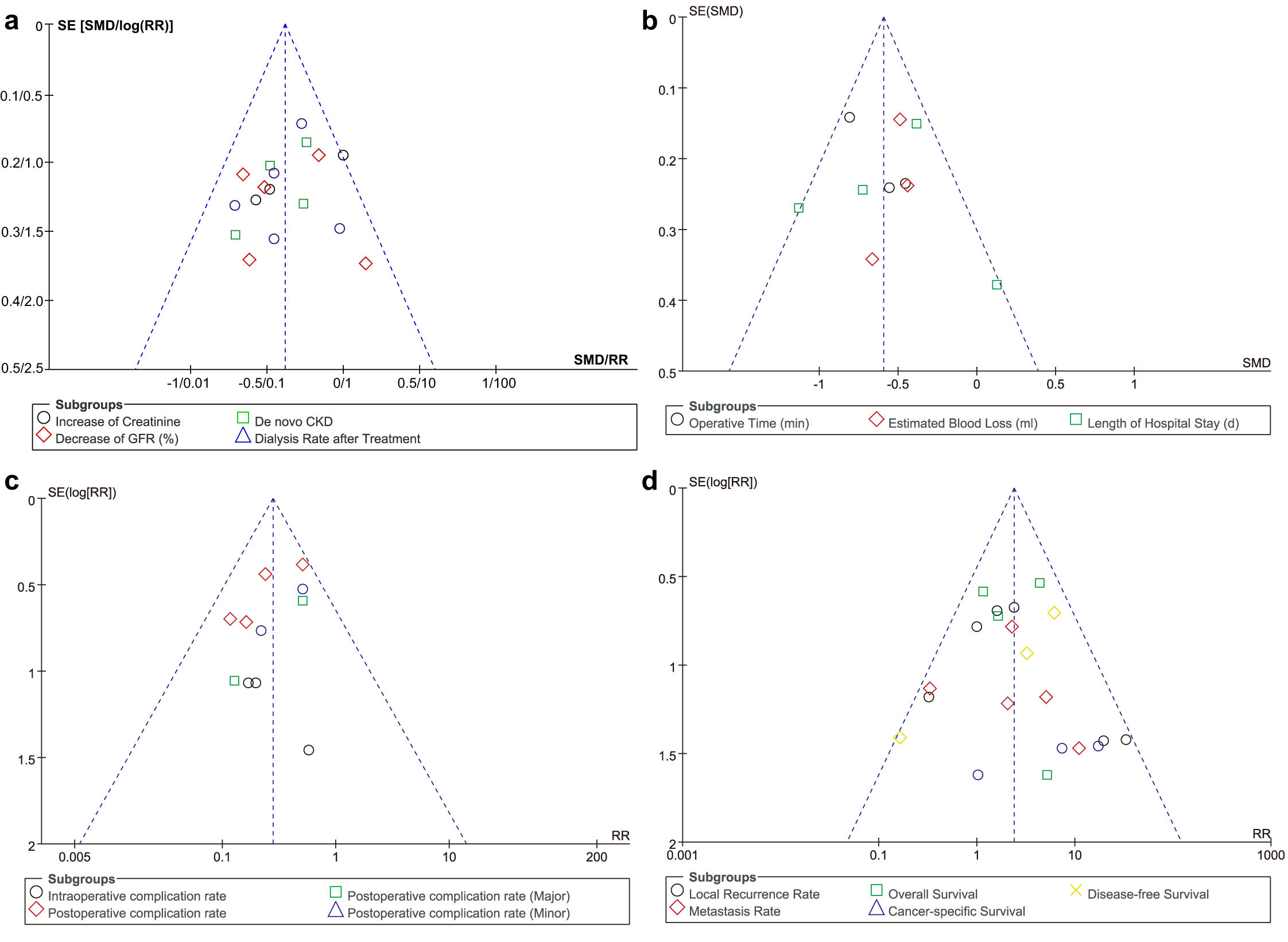

Supplement: S4 Fig — Funnel plots illustrating meta-analysis of renal function outcomes (a), oncologic outcomes (b), perioperative outcomes (c), and complications (d). SE = standard error; SMD = standard mean difference; RR = risk ratio. (TIF) [file pone.0131290.s004.tif]
