# Supplementary material for: Safety and Efficacy of Thermal Ablation for Small Renal Masses in Solitary Kidney: Evidence from Meta-Analysis of Comparative Studies
Source: PLoS One. 2015 Jun 29;10(6):e0131290. doi: 10.1371/journal.pone.0131290 (PMC4484808; doi:10.1371/journal.pone.0131290)
Supplement: S1 File — (DOC) [file pone.0131290.s005.doc]

**Appendix A**

| #1 "Carcinoma, Renal Cell"[Mesh]  #2 "Kidney cancer"  #3 "Renal cancer"  #4 "Kidney tumor"  #5 "Renal mass"  #6 "RCC"  #7 #1 OR #2 OR #3 OR #4 OR #5 OR #6  #8 "Ablation Techniques"[Mesh]  #9 "Cryoablation"  #10 "Ablation"  #11 "Radiofrequency ablation"  #12 "Ablative Therapy"  #13 #8 OR #9 OR #10 OR #11 OR #12  #14 "Partial Nephrectomy"  #15 "Nephron-sparing surgery"  #16 "NSS"  #17 "Enucleation"  #18 #14 OR #15 OR #16 OR #17  #19 #7 AND #13 AND #18 |
| --- |
