# Supplementary material for: Safety and Efficacy of Thermal Ablation for Small Renal Masses in Solitary Kidney: Evidence from Meta-Analysis of Comparative Studies
Source: PLoS One. 2015 Jun 29;10(6):e0131290. doi: 10.1371/journal.pone.0131290 (PMC4484808; doi:10.1371/journal.pone.0131290)
Supplement: S2 File — (DOC) [file pone.0131290.s006.doc]

**Appendix B**

Modified Newcastle-Ottawa scale

**Selection**

1. Assignment for treatment (Details of criteria for adequate random assignment of patients to treatments were provided).

2. Representative of treatment group.

3. Representative of control group.

4. Selection of contemporary series of controls.

**Comparability**

5. Study controls for age, gender and body mass index (one star can be got if only the both characteristics are comparable).

6. Study controls for

1) Preoperative renal function (Creatinine, eGFR and percent of CKD);

2) Number, side and size of renal masses, as well as their pathology;

3) American Society of Anesthesiologists score and severity of comorbidity;

If more than one characteristics were comparable, two stars; if only one characteristic was comparable, one star; otherwise, no star.

**Outcome**

7. Ascertainment of outcome

8. Adequate follow-up
