# Supplementary material for: Safety and Efficacy of Thermal Ablation for Small Renal Masses in Solitary Kidney: Evidence from Meta-Analysis of Comparative Studies
Source: PLoS One. 2015 Jun 29;10(6):e0131290. doi: 10.1371/journal.pone.0131290 (PMC4484808; doi:10.1371/journal.pone.0131290)
Supplement: S3 File — (DOC) [file pone.0131290.s007.doc]

**Appendix C**

[1] Bensalah K, Zeltser I, Tuncel A, et al. Evaluation of costs and morbidity associated with laparoscopic radiofrequency ablation and laparoscopic partial nephrectomy for treating small renal tumours. BJU Int 2008;101:467-71.

[2] Bird VG, Carey RI, Ayyathurai R, et al. Management of renal masses with laparoscopic-guided radiofrequency ablation versus laparoscopic partial nephrectomy. J Endourol 2009;23:81-8.

[3] Desai MM, Aron M, Gill IS. Laparoscopic partial nephrectomy versus laparoscopic cryoablation for the small renal tumor. Urology 2005;66:23-8.

[4] Guan W, Bai J, Liu J, et al. Microwave ablation versus partial nephrectomy for small renal tumors: intermediate-term results. J Surg Oncol 2012;106:316-21.

[5] Guillotreau J, Haber GP, Autorino R, et al. Robotic partial nephrectomy versus laparoscopic cryoablation for the small renal mass. Eur Urol 2012;61:899-904.

[6] Haramis G, Graversen JA, Mues AC, et al. Retrospective comparison of laparoscopic partial nephrectomy versus laparoscopic renal cryoablation for small (<3.5 cm) cortical renal masses. J Laparoendosc Adv Surg Tech A 2012;22:152-7.

[7] Kiriluk KJ, Shikanov SA, Steinberg GD, et al. Laparoscopic partial nephrectomy versus laparoscopic ablative therapy: a comparison of surgical and functional outcomes in a matched control study. J Endourol 2011;25:1867-72.

[8] Klatte T, Mauermann J, Heinz-Peer G, et al. Perioperative, oncologic, and functional outcomes of laparoscopic renal cryoablation and open partial nephrectomy: a matched pair analysis. J Endourol 2011;25:991-7.

[9] Ko YH, Park HS, Moon DG, et al. A matched-cohort comparison of laparoscopic renal cryoablation using ultra-thin cryoprobes with open partial nephrectomy for the treatment of small renal cell carcinoma. Cancer Res Treat 2008;40:184-9.

[10] Lian HB, Guo HQ, Gan WD, et al. [A retrospective study comparing the clinical efficacy of laparoscopic cryoablation and partial nephrectomy for renal cell carcinoma]. Zhonghua Wai Ke Za Zhi 2010;48:834-7.

[11] Lin YC, Turna B, Frota R, et al. Laparoscopic partial nephrectomy versus laparoscopic cryoablation for multiple ipsilateral renal tumors. Eur Urol 2008;53:1210-6.

[12] Lucas SM, Stern JM, Adibi M, et al. Renal function outcomes in patients treated for renal masses smaller than 4 cm by ablative and extirpative techniques. J Urol 2008;179:75-9, 79-80.

[13] O'Malley RL, Berger AD, Kanofsky JA, et al. A matched-cohort comparison of laparoscopic cryoablation and laparoscopic partial nephrectomy for treating renal masses. BJU Int 2007;99:395-8.

[14] Stern JM, Svatek R, Park S, et al. Intermediate comparison of partial nephrectomy and radiofrequency ablation for clinical T1a renal tumours. BJU Int 2007;100:287-90.

[15] Sung HH, Park BK, Kim CK, et al. Comparison of percutaneous radiofrequency ablation and open partial nephrectomy for the treatment of size- and location-matched renal masses. Int J Hyperthermia 2012;28:227-34.

[16] Takaki H, Yamakado K, Soga N, et al. Midterm results of radiofrequency ablation versus nephrectomy for T1a renal cell carcinoma. Jpn J Radiol 2010;28:460-8.

[17] Whitson JM, Harris CR, Meng MV. Population-based comparative effectiveness of nephron-sparing surgery vs ablation for small renal masses. BJU Int 2012;110:1438-43, 1443.

[18] Choueiri TK, Schutz FA, Hevelone ND, et al. Thermal ablation vs surgery for localized kidney cancer: a Surveillance, Epidemiology, and End Results (SEER) database analysis. Urology 2011;78:93-8.

[19] Panumatrassamee K, Kaouk JH, Autorino R, et al. Cryoablation versus minimally invasive partial nephrectomy for small renal masses in the solitary kidney: impact of approach on functional outcomes. J Urol 2013;189:818-22.
