# Supplementary material for: Safety and Efficacy of Thermal Ablation for Small Renal Masses in Solitary Kidney: Evidence from Meta-Analysis of Comparative Studies
Source: PLoS One. 2015 Jun 29;10(6):e0131290. doi: 10.1371/journal.pone.0131290 (PMC4484808; doi:10.1371/journal.pone.0131290)
Supplement: S1 Table — (DOC) [file pone.0131290.s008.doc]

Table S1-Risk of bias in retrospective studies using modified Newcastle-Ottawa scale

| Study | Selection | | | | Comparability | | Outcome | | Quality score |
| --- | --- | --- | --- | --- | --- | --- | --- | --- | --- |
|  | 1 | 2 | 3 | 4 | 5 | 6 | 7 | 8 |  |
| Mues 2012 | No | Yes | Yes | Yes | Yes | Yes | Yes | Yes | ★★★★★★★ |
| Kamol 2013 | No | Yes | Yes | No | Yes | Yes | Yes | Yes | ★★★★★★ |
| Turna 2009 | No | Yes | Yes | No | Yes | Yes | Yes | No | ★★★★★★ |
| Goyal 2011 | No | Yes | Yes | Yes | Yes | Yes | Yes | Yes | ★★★★★★★ |
| Mitchell 2011 | No | Yes | Yes | Unclear | Yes | Yes | Yes | Yes | ★★★★★★ |
| Raman 2009 | No | Yes | Yes | No | No | No | Yes | Yes | ★★★★ |
| Olweny 2012 | No | Yes | Yes | Yes | No | Yes | Yes | Yes | ★★★★★★★ |
| Haber 2011 | No | Yes | Yes | No | Yes | Yes | Yes | Yes | ★★★★★★★ |
