# Supplementary material for: Safety and Efficacy of Thermal Ablation for Small Renal Masses in Solitary Kidney: Evidence from Meta-Analysis of Comparative Studies
Source: PLoS One. 2015 Jun 29;10(6):e0131290. doi: 10.1371/journal.pone.0131290 (PMC4484808; doi:10.1371/journal.pone.0131290)
Supplement: S1 PRISMA Checklist — (DOC) [file pone.0131290.s009.doc]

| **Section/topic** | **#** | **Checklist item** | **Reported on page #** |
| --- | --- | --- | --- |
| **TITLE** | | |  |
| Title | 1 | Safety and Efficacy of Thermal ablation for Small Renal Masses in solitary kidney: Evidence from Meta-analysis of Comparative Studies | 1 |
| **ABSTRACT** | | |  |
| Structured summary | 2 | OBJECTIVE: To evaluate the renal functional preservation, oncologic outcomes, perioperative outcomes, and complications for TA versus partial nephrectomy (PN) in management of SRMs in a solitary kidney.  METHODS AND FINDINGS: Medline, Embase, Web of Science and the Cochrane Library were systematically searched. A meta-analysis of comparative studies comparing TA with PN was performed. According to predefined inclusion criteria, seven distinct datasets from 8 observational studies including a total of 628 patients were identiﬁed. Cumulated data showed that the changes of creatinine (p=0.02) and estimated glomerular ﬁltration rate (eGFR) (p<0.0001) in the TA arm were both significantly smaller than these of the PN arm. Significantly different incidence of new-set chronic kidney disease (CKD) favoring TA was observed (p=0.04). In terms of postoperative dialysis rate, the difference favoring TA was also noted, though there is no statistical significance (p=0.09). With regard to perioperative outcomes, our data demonstrated that patients who underwent TA had significantly shorter operation time (p=0.002), less blood loss (p<0.0001), shorter length of stay (p<0.00001), and less transfusion rate (p=0.01) than those underwent PN. In addition, patients underwent TA suffered less intra- and postoperative complications (p=0.007, p<0.00001; respectively). With regard to oncologic outcomes, TA yielded a comparable overall survival to PN (p=0.40). But cumulative analysis revealed that disease-free survival (DFS) (p<0.00001) and cancer-specific survival (CSS) (p=0.01) in the PN arm were significantly higher than these of the TA arm. Additionally, Estimate of the local recurrence rate in those patients treated with TA was in a similar direction with DFS and CSS, but it did not reach signiﬁcance (p=0.15). Sensitivity analyses led to very similar results with overall results, and confirmed its stability.  CONCLUSIONS: Our data suggest that indication for TA may be extended to select younger, healthier patients who desire a much less invasive therapeutic option. | 2 |
| **INTRODUCTION** | | |  |
| Rationale | 3 | In recent years, a number of studies have been published in an attempt to explore if the TA is an effective and safe substitution for treatment of SRMs in solitary kidney, but the results are inconsistent. In addition, small sample size at single-centre prevents credible conclusions from being obtained. Therefore, this systematic review and meta-analysis of comparative studies was performed. | 4 |
| Objectives | 4 | To evaluate the renal functional preservation, oncologic outcomes, perioperative outcomes, and complications for TA versus partial nephrectomy (PN) in management of SRMs in a solitary kidney. | 4 |
| **METHODS** | | |  |
| Protocol and registration | 5 | Comprehensive literature review was conducted by a search of Medline, Embase, Web of Science and the Cochrane Library using predefined search terms (Appendix A) without language and date restriction. We also reviewed the reference lists of relevant publications. Only full-text articles published in peer-reviewed journals were identified. No registration. | 5 |
| Eligibility criteria | 6 | 1) Patients with functional or anatomic solitary kidney were evaluated. 2) Comparative data were available. Cases were treated with CA, RFA or other ablation techniques, while subjects from control group were treated with methods of PN or enucleation. 3) Original data for dichotomous and/or continuous variables had to be provided or calculable from the data source. 4) When some studies with the same population were identified, all of them were assessed carefully to ensure no evidence was missed, so more than one of them might be included together. | 5 |
| Information sources | 7 | References of included studies and narrative reviews were searched for potential studies. | 5 |
| Search | 8 | Comprehensive literature review was conducted by a search of Medline, Embase, Web of Science and the Cochrane Library using predefined search terms (Appendix A) without language and date restriction. Database up to December 2014. | 5 |
| Study selection | 9 | Included studies were restricted to studies that should meet all of the following inclusion criteria: 1) Patients with functional or anatomic solitary kidney were evaluated. 2) Comparative data were available. Cases were treated with CA, RFA or other ablation techniques, while subjects from control group were treated with methods of PN or enucleation. 3) Original data for dichotomous and/or continuous variables had to be provided or calculable from the data source. 4) When some studies with the same population were identified, all of them were assessed carefully to ensure no evidence was missed, so more than one of them might be included together. | 5 |
| Data collection process | 10 | Two investigators (Wang and Nie) independently extracted data, and disagreements were resolved through discussion with a third author (Yang). | 6 |
| Data items | 11 | The listed study characteristics were considered: study design, numbers of cases and control subjects, gender, age, Body Mass Index (BMI), comorbidity, American Society of Anesthesiologists (ASA) score, preoperative renal function and its change, and the side, size and pathology of masses. | 6 |
| Risk of bias in individual studies | 12 | The methodological quality of retrospective studies was assessed by the modified Newcastle-Ottawa scale [19], which consists of three elements: selection of subjects, comparability of the study groups, and assessment of outcome (Appendix C). A score of 0–9 was allocated to each study, and observational studies achieving six or more stars were considered to be of high quality. | 6 |
| Summary measures | 13 | The main outcomes were renal functional outcomes. Four measurements on renal function were evaluated including changes of creatinine and percent of estimated glomerular ﬁltration rate (eGFR), new-set chronic kidney disease (CKD) and postoperative dialysis rate (percent of patients need dialysis after treatment). We take into account all patients requiring temporary or persistent dialysis. Subgroup analysis for persistent dialysis rate wasn’t addressed because of insufficient data. The secondary outcomes were perioperative outcomes (e.g., operative time, estimated blood loss [EBL], length of stay [LOS], transfusion rate and conversion rate), complications (intraoperative complications and postoperative complications within 30 d of surgery) and oncologic outcomes (local recurrence rate, metastasis rate, overall survival [OS], cancer-specific survival [CSS] and recurrence-free survival [RFS]). If sufficient data were available, postoperative complications were subdivided into major complications and minor complications. Postoperative complications were classified according to the Clavien classiﬁcation system [20]. Minor complications were deﬁned as grade 1 and 2, and major complications included grades 3–5. Local recurrence was radiologically defined as tumor relapsed or newly observed in kidney during the follow-up period. | 6 |
| Synthesis of results | 14 | Patients with worse renal function might be likely selected to be treated with TA [14,15]. So, one-point (such as postoperative) creatinine and eGFR may bias us against TA when evaluating renal functional outcomes. As a result, the increase of creatinine, decrease of eGFR, incidence of new-set CKD and postoperative dialysis rate were cumulated in this analysis. Cumulated data showed that the increase of creatinine (MD: -0.14; 95% CI, -0.26 to -0.03; p=0.02; Fig. 2a) and decrease of eGFR (MD: -9.84; 95% CI, -14.25 to -5.44; p<0.0001; Fig. 2b) in the TA arm were both significantly fewer than these in PN arm. Significantly different incidence of new-set CKD favoring TA was also observed (RR: 0.44; 95% CI, 0.20-0.95; p=0.04; Fig. 2c). For postoperative dialysis rate, a non-statistically significant difference favoring TA was noted (RR: 0.38; 95% CI, 0.13-1.16; p=0.09; Fig. 2e). We failed to find any notable between-study heterogeneity (p=0.12, I2=52%; p=0.43, I2=0%; p=0.79, I2=0%; p=0.93, I2=0%; respectively), which shows favorable internal consistency among included studies. | 7 |

Page 1 of 2

| **Section/topic** | **#** | **Checklist item** | **Reported on page #** |
| --- | --- | --- | --- |
| Risk of bias across studies | 15 | All the studies included were observational studies with no randomization. Study quality ratings on some properties were too skewed to enable estimation of inter-rater reliability. Absolute agreement across properties ranged from four to seven of nine stars. Only one study reached less than six stars and was identified as of low quality. After eliminating this study, sensitivity analysis was conducted to confirm the reliability of our findings. | Table S1 |
| Additional analyses | 16 | Six high-quality retrospective studies [9-14,16] scored six or more stars on the modified Newcastle-Ottawa scale were included in the sensitivity analyses. These led to very similar results with overall analyses. Only one change in the significance was observed in the cumulated outcomes evaluating incidence of new-set CKD after treatment (Fig. 2d). Nevertheless, persistent trend favoring TA still existed (RR: 0.52; 95% CI, 0.24-1.14; p=0.10). In our assessment of publication bias, funnel plots showed balance, with points distributing around the vertical, indicating no obvious publication bias existed (Fig. S4). | 7 |
| **RESULTS** | | |  |
| Study selection | 17 | We screened 378 potentially relevant, non-duplicate articles. In light of titles and abstracts, 109 papers with full texts might be of potential interest were got. After reading through full-texts, 28 publications that appeared to meet the inclusion criteria were subjected to further examination. Among them, 19 records were excluded because no data for solitary kidney was available (Appendix B). The ﬁnal number of papers included in the meta-analysis was 8 [9-16], which reported on 7 distinct datasets with 324 cases and 304 controls (Fig. 1). We suspected that four articles [10,14,16,24] had overlapping datasets but three of them [10,14,16] presented data on different outcomes, therefore, we only excluded one [24]. In addition, one article reported two separate datasets, one for CA versus LPN (named a) and one for RFA versus LPN (named b) [16].. | 8 |
| Study characteristics | 18 | Details of the included studies were summarized in Table 1. | Table 1 |
| Risk of bias within studies | 19 | In our assessment of publication bias, funnel plots showed balance, with points distributing around the verticals, indicating no obvious publication bias (see Web-Figure 2). Additionally, actualized data from Begg’s and Egger’s tests also suppoted no exhibited publication bias (see Table 2). | 12 |
| Results of individual studies | 20 | For all outcomes considered (benefits or harms), present, for each study: (a) simple summary data for each intervention group (b) effect estimates and confidence intervals, ideally with a forest plot. | 8 |
| Synthesis of results | 21 | Our analysis indicates that PN have advantage in controlling cancer recurrence. However, TA is associated with significantly better renal functional preservation and perioperative outcomes and fewer complications without increasing overall death. More kidney cancer–related deaths with TA tended to be balanced by more deaths not related to prostate cancer with PN. | 8 |
| Risk of bias across studies | 22 | In our assessment of publication bias, funnel plots showed balance, with points distributing around the vertical, indicating no obvious publication bias existed | 12 |
| Additional analysis | 23 | Six high-quality retrospective studies [9-14,16] scored six or more stars on the modified Newcastle-Ottawa scale were included in the sensitivity analyses. These led to very similar results with overall analyses. Only one change in the significance was observed in the cumulated outcomes evaluating incidence of new-set CKD after treatment (Fig. 2d). Nevertheless, persistent trend favoring TA still existed (RR: 0.52; 95% CI, 0.24-1.14; p=0.10). | 12 |
| **DISCUSSION** | | |  |
| Summary of evidence | 24 | Because of the rare incidence of SRMs in solitary kidney, we failed to carry out a randomized-controlled trial (RCT) with long term follow up to compare TA with PN concerning renal functional preservation, morbidity, and cancer control. Then this meta-analysis based on seven observational studies involving 324 cases and 304 controls is conducted. Our analysis indicates that PN have advantage in controlling cancer recurrence. However, TA is associated with significantly better renal functional preservation and perioperative outcomes and fewer complications without increasing overall death. More kidney cancer–related deaths with TA tended to be balanced by more deaths not related to prostate cancer with PN. | 12 |
| Limitations | 25 | First limitation is that all studies included in our analysis were retrospective, and that may introduced the potential bias for selection. Patients undergoing TA might be accompanied with significant comorbidities [33], indicating that observed difference of renal function and complications favoring TA might be weakened. Nevertheless, small exophytic tumors have tended to be treated with TA while large and complex tumors are usually managed with PN [33], which may causes overestimating the advantages of TA on complications and perioperative outcomes. Unfortunately, data to stratify tumors by complexity were insufficient for analysis. Another main limitation is the small sample sizes of included studies in this meta-analysis, which is a reflection of the rarity of SRMs in solitary kidney. Finally, the follow-up periods were generally short and different between the comparative groups, so outcomes with identical and long term follow-up, especially for oncologic and renal functional outcomes, are expected. | 17 |
| Conclusions | 26 | For patients with a solitary kidney, PN and TA are both safe and effective treatment options. PN is superior to TA in controlling tumor recurrence and death caused by cancer. Compared to PN, TA is associated with better renal functional maintenance, shorter operation time, less EBL, shorter LOS, and less intra- and postoperative complications. As an effective minimally invasive therapy, TA yields an equivalent long-term OS to PN. These results suggest that indication for TA may be extended to select younger, healthier patients who desire a much less invasive therapeutic option. Further well-designed RCTs with extensive follow-up are needed to confirm and update our findings. | 18 |
| **FUNDING** | | |  |
| Funding | 27 | The work was not supported by funding. | 18 |

*From:*  Moher D, Liberati A, Tetzlaff J, Altman DG, The PRISMA Group (2009). Preferred Reporting Items for Systematic Reviews and Meta-Analyses: The PRISMA Statement. PLoS Med 6(6): e1000097. doi:10.1371/journal.pmed1000097

For more information, visit: **www.prisma-statement.org**.

Page 2 of 2
